# Early immunological responses to the mRNA SARS-CoV-2 vaccine in patients with neuromuscular disorders

**DOI:** 10.3389/fimmu.2022.996134

**Published:** 2022-09-29

**Authors:** Hideyuki Iwayama, Naoko Ishihara, Kohei Kawahara, Yuta Madokoro, Yasuko Togawa, Kanji Muramatsu, Ayuka Murakami, Satoshi Kuru, Toshiyuki Kumagai, Wataru Ohashi, Kengo Nanya, Shinji Hasegawa, Masahisa Katsuno, Akihisa Okumura

**Affiliations:** ^1^ Department of Pediatrics, Aichi Medical University School of Medicine, Nagakute, Japan; ^2^ Department of Pediatrics, Fujita Health University School of Medicine, Toyoake, Japan; ^3^ Department of Neurology, Nagoya City University Hospital, Nagoya, Japan; ^4^ Department of Pediatrics, Toyohashi Municipal Hospital, Toyohashi, Japan; ^5^ Department of Neurology, National Hospital Organization Suzuka Hospital, Suzuka, Japan; ^6^ Department of Neurology, Nagoya University Graduate School of Medicine, Nagoya, Japan; ^7^ Department of Pediatric Neurology, Aichi Prefectural Colony Central Hospital, Kasugai, Japan; ^8^ Kuma Home Medical Care Clinic, Nagoya, Japan; ^9^ Division of Biostatistics, Clinical Research Center, Aichi Medical University School of Medicine, Nagakute, Japan; ^10^ Clinical Laboratory, Nagoya Memorial Hospital, Nagoya, Japan; ^11^ Department of Pediatrics, Nagoya Memorial Hospital, Nagoya, Japan; ^12^ Department of Clinical Research Education, Nagoya University Graduate School of Medicine, Nagoya, Japan

**Keywords:** neuromuscular disorders, SARS-CoV-2, vaccination, spinal muscular atrophy, Duchenne muscular dystrophy, residual muscle volume

## Abstract

**Backgrounds:**

Intramuscular injection of the SARS-CoV-2 vaccine has raised concerns about its use in patients with neuromuscular disorders (NMDs). We evaluated the response of patients with NMDs to the BNT162b2 vaccine.

**Methods:**

Healthy subjects, patients with spinal muscular atrophy (SMA), and patients with Duchenne muscular dystrophy (DMD) were included. All participants received two BNT162b2 doses. SARS-CoV-2 antibody titers at baseline and 2 weeks after each vaccination were compared between groups. Residual muscle volume was evaluated in NMDs group. A questionnaire documented adverse reactions.

**Results:**

Eleven patients with NMDs (9 with SMA, 2 with DMD; 7 males; aged 32.7 ± 19.3 years) and 346 healthy subjects (60 males, aged 40.0 ± 12.4 years) were included. Antibody titers (U/mL) were similar between groups (baseline: <0.40 vs. <0.40, first vaccination, 145 ± 258 vs. 103 ± 1192, and second vaccination, 1528 ± 1265 vs. 1429 ± 944; p = 1.000, 0.909, and 0.736, respectively). A negative correlation was found between antibody titers and residual muscle volume but was not significant (Mercuri scale, r = −0.429, p = 0.249; fat infiltration rate, r = −0.194, p = 0.618). The adverse reactions were comparable between groups.

**Conclusion:**

The BNT162b2 vaccine is safe and effective in patients with NMDs.

## Introduction

Coronavirus-2019 (COVID-19) is caused by the severe acute respiratory syndrome coronavirus-2 (SARS-CoV-2). BNT162b2 is an mRNA-based SARS-CoV-2 vaccine manufactured by Pfizer (1). However, because BNT162b2 is given by intramuscular injection, there is concern about its safety and efficacy in patients with neuromuscular disorders (NMDs).

Spinal muscular atrophy (SMA) and Duchenne muscular dystrophy (DMD) are rare inherited NMDs that cause muscular atrophy. While individuals with NMDs who are not taking immunosuppressive agents are currently encouraged to receive COVID-19 vaccines (1), only one study has reported the efficacy and safety of the vaccine in these patients (2).

In this study, we aimed to evaluate the safety and efficacy of BNT162b2 in patients with NMDs with muscular atrophy compared to that in healthy subjects. We also examined whether residual muscle volume affects serum levels of the SARS-CoV-2 antibody titer.

## Subjects and methods

### Ethical compliance

This multi-center prospective observational cohort study was performed in Japan. It was approved by the ethics committee of Aichi Medical University (approval no. 2021-075). All participants provided written informed consent.

### Subjects

Employees of Nagoya Memorial Hospital aged 21 years or older were included as healthy subjects (control group). Adolescents or adults aged 12 years or older with SMA or DMD with muscular atrophy who were wheelchair-bound or bedridden were included as patients with NMDs (NMDs group). Diagnoses of NMDs were performed by genetic testing. All participants received two BNT162b2 injections into the deltoid muscle between March and August 2021. We had selected the deltoid muscle because Ministry of Health, Labour and Welfare of Japan recommend that the preferred site was the deltoid muscle of the upper arm (3). Blood was collected from all participants at baseline and two weeks after each dose of the vaccine. Patients with a history of COVID-19 infection or elevated antibody titers prior to vaccination were excluded.

### Evaluation

A quantitative determination of antibodies against the receptor binding domain of the SARS-CoV-2 S1 subunit of the spike protein was made using plasma samples at baseline and two weeks after each dose of BNT162b2 (Elecsys anti-SARS-CoV-2 S, Roche Diagnostics International Ltd., Rotkreuz, Switzerland), as previously reported (4). Residual muscle volume was assessed by computed tomography (CT) within 1 year before and after the vaccination date. A slice of CT from the humeral head was obtained to assess the atrophy of the deltoid muscle of each patient using the Mercuri scale (5). CT scans were performed for periodic evaluation of scoliosis and chest deformities, not for the study purpose. We did not select to evaluate the muscular atrophy with echo examination, because we were not familiar with the evaluation of the muscular atrophy with echo examination. The images were blindly evaluated by one reviewer (HI). The muscle and fat were circled using Image J software v. 1.53m (National Institutes of Health) and the each area was measured, as previously reported (6). Fat infiltration rate (FIR) was calculated by dividing the fatty area (cm^2^) by the total area of the deltoid muscle (cm^2^). Information on adverse reactions was obtained from participants by questionnaire and classified using the Common Terminology Criteria for Adverse Events v. 4.0. All patients answered the side effect by themselves, not family members. They were not diagnosed with intellectual disability. If the information was missing, the subject was excluded from the evaluation of adverse reactions.

### Statistical analysis

Data were expressed as mean ± standard deviation. Qualitative variables were compared using Pearson’s chi-squared tests. The numerical variables in each group, including antibody titers, were analyzed by student t-tests. The correlations between antibody titers and Mercuri scale and antibody titers and FIR were determined using Pearson correlation tests. The significance level was defined as *p* < 0.05.

All statistical analyses were performed using EZR (Saitama Medical Center, Jichi Medical University, Saitama, Japan) (7), which is a modified version of R commander, designed to perform statistical functions frequently employed in biostatistics (The R Foundation for Statistical Computing, Vienna, Austria).

## Results

### Participants

Of the participants, 50 subjects in the healthy control group and one patient with NMDs were excluded due to a history of COVID-19 infection or elevated antibody titers prior to vaccination. Eleven patients with NMDs (SMA, 9; DMD, 2. M: F = 7: 4) and 346 healthy subjects (M: F = 60: 286) were included in the study (**Table 1**). Seven patients with SMA and two patients with DMD were being treated with nusinersen and viltolarsen, respectively (**Table 2**). No patients with NMD took any oral steroids or immunosuppressive drugs. CT scans were obtained from nine patients with NMDs. The Mercuri scale grades of the NMDs group were grade 2b (n = 1), grade 3 (n = 4), and grade 4 (n = 4), with a mean FIR of 85.2 ± 16.6%.

**Table 1:** Background and SARS-CoV-2 antibody titers of the control and NMDs groups.

Variables	Control group	NMDs group	*p* value
N	346	11 (SMA, 9; DMD, 2)	
Sex (male:female)	60:286	7:4	0.001
Age (years)	40.0 ± 12.4	32.7 ± 19.3	0.062
SARS-CoV-2 antibody titers (U/mL)			
Before vaccination	<0.40	<0.40	1.000
Two weeks after first vaccination	103 ± 1192	145 ± 258	0.909
Male	26 ± 29	203 ± 312	<0.001
Female	42 ± 69	42 ± 70	0.998
Two weeks after second vaccination	1429 ± 944	1528 ± 1265	0.736
Male	1167 ± 633	1206 ±990	0.886
Female	1486 ± 991^*^	2091 ±1646	0.230

DMD: Duchenne muscular dystrophy; NMDs: neuromuscular disorders; SMA: spinal muscular atrophy; *, In the control group, females had significantly higher antibody titers than males after the second vaccination (p = 0.0176).

**Table 2:** Characteristics and residual muscle volume of NMD patients.

No.	Age	Sex	Disease	Treatment	Mercuri scale	Fat infiltration rate (%)
1	29	M	SMA2	Nusinersen	4	100
2	26	F	SMA2	Nusinersen	3	76.3
3	23	M	SMA2	Nusinersen	4	100
4	42	M	SMA2	Nusinersen	4	100
5	23	F	SMA2	ND	ND	ND
6	17	M	DMD	Viltolarsen	2b	59.4
7	20	M	DMD	Viltolarsen	3	61.5
8	16	F	SMA1	ND	4	100
9	65	M	SMA3	Nusinersen	3	87.8
10	73	M	SMA3	ND	3	82.0
11	12	F	SMA1	Nusinersen	ND	ND

DMD, Duchenne muscular dystroph; ND, not done; NMDs, neuromuscular disorders; SMA, spinal muscular atrophy.

### Antibody titers obtained against BNT162b2

**Figure 1:**
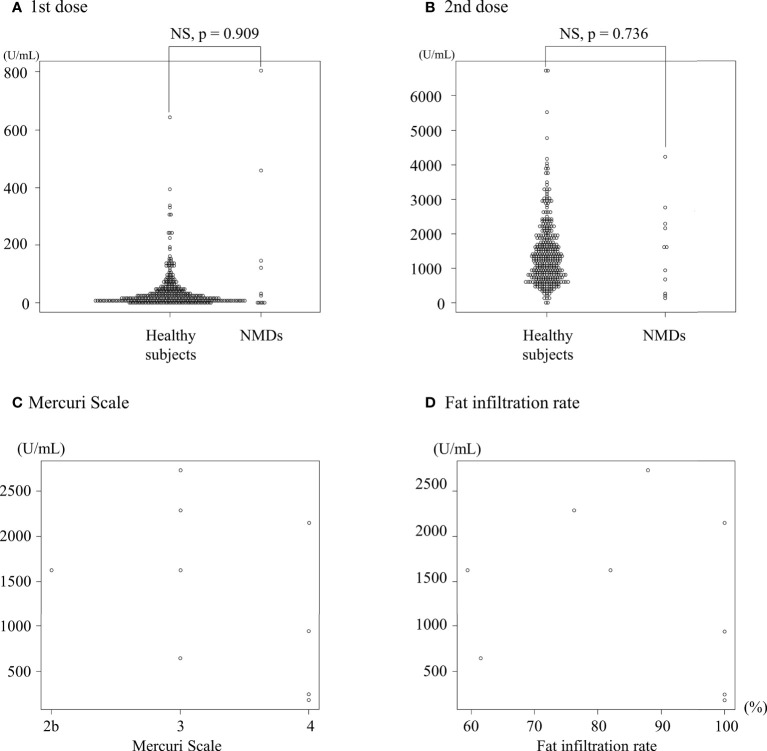
SARS-CoV-2 antibody titers after vaccination. SARS-CoV-2 antibody titers two weeks after the first **(A)** and second **(B)** dose of vaccination in　healthy controls (n = 346) and patients with neuromuscular disorders (NMDs) (n = 11). Fat infiltration in the deltoid muscle on the Mercuri Scale **(C)** and fat infiltration rate **(D)**, and SARS-CoV-2 antibody titers after the second dose of vaccination in patients with NMDs.

Antibody titers (U/mL) were similar between the NMDs and control groups (baseline: <0.40 vs. <0.40, first vaccination, 145 ± 258 vs. 103 ± 1192, and second vaccination, 1528 ± 1265 vs. 1429 ± 944; *p* = 1.000, 0.909, and 0.736, respectively) (**Table 1**, **Figures 1A, B**). Although a negative correlation was seen between antibody titers and residual muscle volume, it was not statistically significant either on the Mercuri scale (r = −0.429, *p* = 0.249) or FIR (r = −0.194, *p* = 0.618). (**Figures 1C, D**). The relationship between the antibody titers two weeks after the first and second vaccination in the NMD group was evaluated, but no correlation was found (r = 0.0744, *p* = 0.828).

When splitting the responses into male and female response, antibody titers two weeks after the first vaccination in males were significantly higher in the NMDs group than in the control group (203 ± 312 vs. 26 ± 29, *p* < 0.001) (**Table 1**). Antibody titers two weeks after the first vaccination in females were not significantly different between groups (control: 42 ± 69 vs. NMDs: 42 ± 70, *p* = 0.998) (**Table 1**). Antibody titers two weeks after the second vaccination were not significantly different between healthy and NMD groups for either males or females (M: 1167 ± 633 vs. 1206 ± 990, *p* = 0.886; F: 1486 ± 991 vs. 2091 ± 1646, *p* = 0.230) (**Table 1**).

In the control group, females had significantly higher antibody titers than males after the second vaccination (1st vaccination, M: 26 ± 29 vs. F: 42 ± 69, *p* = 0.0779; 2nd vaccination, M: 1167 ± 633 vs. F: 1486 ± 991, *p* = 0.0176),but in the NMD group, female patients did not have significantly higher antibody titers than male patients after either the first or second vaccination (1st vaccination, M: 203 ± 312 vs. F: 42 ± 70, *p* = 0.345; 2nd vaccination, M: 1206 ± 990 vs. F: 2091 ± 1646, *p* = 0.287).

### Safety of and adverse reactions caused by BNT162b2

Due to lack of information, seven of the healthy control subjects were excluded from the evaluation of adverse reactions (**Table 3**). No anaphylaxis occurred in any of the participants. Vasovagal reflex occurred in one participant in the control group after the second vaccination. The frequency of any adverse reactions ≥ grade 1 was lower in the NMDs group than in the control group after each dose of the vaccine, but the frequency of any adverse reactions ≥ grade 2 or 3 was comparable between the groups (first vaccination: grade 1, *p* = 0.0007; grade 2, *p* = 0.2524; grade 3, p = 0.685 and second vaccination: grade 1, *p* = 0.0007; grade 2, *p* = 0.9244; grade 3, *p* = 1.000) (**Table 3**). The frequency of injection site reaction was similar in both the groups during the first vaccination; however, it was lower in the NMDs group than in the control group during the second vaccination (first vaccination, *p* = 0.3484; second vaccination, *p <*0.0001). Other side effects and grades were similar among the groups. Furthermore, the frequency of analgesic use was similar in both the groups during the first vaccination, whereas it was lower in the NMDs group than in the control group during the second vaccination (first vaccination, *p* = 0.3019; second vaccination, *p* = 0.0252).

**Table 3:** Adverse reactions observed in the control and NMD groups.

	After the first vaccination			After the second vaccination		
Adverse reactions	Control group (n = 339)	NMDs group (n = 11)	*p* value	Control group (n = 339)	NMDs group (n = 11)	*p* value
Any adverse reactions						
≥grade 1	329 (97.1%)	8 (72.7%)	0.0007	329 (97.1%)	8 (72.7%)	0.0007
≥grade 2	61 (18.0%)	0 (0%)	0.2524	113 (33.3%)	3 (27.2%)	0.9244
≥grade 3	5 (1.5%)	0 (0%)	1.000	6 (1.8%)	0 (0%)	1.000
Anaphylaxis	0 (0%)	0 (0%)	1.000	0 (0%)	0 (0%)	1.000
Vasovagal reflex	0 (0%)	0 (0%)	1.000	1 (0.3%)	0 (0%)	1.000
Injection site reaction						
≥grade 1	271 (80.0%)	7 (63.6%)	0.3484	310 (91.4%)	5 (45.5%)	<0.0001
≥grade 2	45 (13.3%)	0 (0%)	0.1955	25 (7.4%)	0 (0%)	0.7339
≥grade 3	1 (0.3%)	0 (0%)	1.000	1 (0.3%)	0 (0%)	1.000
Fatigue						
≥grade 1	62 (18.3%)	1 (9.1%)	0.7019	184 (54.3%)	5 (45.5%)	0.7868
≥grade 2	11 (3.2%)	0 (0%)	1.000	65 (19.2%)	2 (18.2%)	1.000
≥grade 3	2 (0.6%)	0 (0%)	1.000	2 (0.6%)	0 (0%)	1.000
Headache						
≥grade 1	54 (15.9%)	1 (9.1%)	0.8474	151 (44.5%)	3 (27.2%)	0.4082
≥grade 2	8 (2.4%)	0 (0%)	1.000	34 (10.0%)	0 (0%)	0.5564
≥grade 3	1 (0.3%)	0 (0%)	1.000	0 (0%)	0 (0%)	1.000
Fever						
≥grade 1	4 (1.2%)	0 (0%)	1.000	71 (20.9%)	1 (9.1%)	0.5631
≥grade 2	3 (0.9%)	0 (0%)	1.000	18 (5.3%)	1 (9.1%)	1.000
≥grade 3	0 (0%)	0 (0%)	1.000	1 (0.3%)	0 (0%)	1.000
Chills						
≥grade 1	3 (0.9%)	0 (0%)	1.000	35 (10.3%)	0 (0%)	0.5401
≥grade 2	0 (0%)	0 (0%)	1.000	22 (6.5%)	0 (0%)	0.8091
Myalgia						
≥grade 1	80 (23.6%)	5 (45.5%)	0.1914	134 (39.5%)	5 (45.5%)	0.9344
≥grade 2	9 (2.7%)	0 (0%)	1.000	24 (7.1%)	0 (0%)	0.7579
≥grade 3	2 (0.6%)	0 (0%)	1.000	2 (0.6%)	0 (0%)	1.000
Arthralgia						
≥grade 1	29 (8.6%)	0 (0%)	0.6475	108 (31.9%)	2 (18.2%)	0.5276
≥grade 2	2 (0.6%)	0 (0%)	1.000	27 (8.0%)	0 (0%)	0.689
≥grade 3	0 (0%)	0 (0%)	1.000	2 (0.6%)	0 (0%)	1.000
Diarrhea						
≥grade 1	13 (3.8%)	0 (0%)	1.000	18 (5.3%)	0 (0%)	0.9274
≥grade 2	2 (0.6%)	0 (0%)	1.000	4 (1.2%)	0 (0%)	1.000
Nausea						
≥grade 1	11 (3.2%)	0 (0%)	1.000	41 (12.1%)	1 (9.1%)	1.000
≥grade 2	5 (1.5%)	0 (0%)	1.000	13 (3.8%)	0 (0%)	1.000
≥grade 3	1 (0.3%)	0 (0%)	1.000	0 (0%)	0 (0%)	1.000
Vomiting						
≥grade 1	7 (2.1%)	0 (0%)	1.000	7 (2.1%)	0 (0%)	1.000
≥grade 2	5 (1.5%)	0 (0%)	1.000	3 (0.9%)	0 (0%)	1.000
≥grade 3	2 (0.6%)	0 (0%)	1.000	0 (0%)	0 (0%)	1.000
Use of analgesics	41 (12.1%)	3 (27.2%)	0.3019	193 (56.9%)	2 (18.2%)	0.0252

NMDs, neuromuscular disorders.

Grades not listed in the table were not observed in patients in either group.

A 73-year-old patient with SMA developed blisters a few days after the first dose of BNT162b2. He was diagnosed with bullous pemphigoid due to the positive BP180 antibody and treated with 15 mg of prednisolone.

## Discussion

This study showed the efficacy and safety of BNT162b2 in patients with NMDs. These patients were found to have antibody titers similar to those of healthy controls after intramuscular vaccination. The frequency of adverse reactions was lower in the NMDs group than in the control group.

### Vaccination in NMDs

As there was no information on mRNA vaccines, the vaccine was administered *via* intramuscular injection in the BNT162b2 trial. Intramuscular administration of vaccines into the deltoid optimizes the immunogenicity of the vaccine and minimizes adverse reactions at the injection site (8). Muscles have good vascularity and therefore allow the injected drug to reach the systemic circulation quickly, bypassing the first-pass metabolism (9). Injecting a vaccine into subcutaneous fat has been reported to cause vaccine failure in hepatitis B, rabies, and influenza vaccines (8). One study compared the immunogenicity of intramuscular versus subcutaneous administration with inactivated influenza vaccine in NMDs (10). There is a review article related to the vaccination in neurological disease, including muscular dystrophy and spinal muscular atrophy (11). However, there were no existing guidelines/recommendations for the vaccination for NMDs.

### Efficacy of BNT162b2 in NMDs

In this study, the antibody titers in the NMDs group were similar to those in the control group two weeks after each dose of the vaccine. Similar to our results, one paper reported increased antibody titers with BNT162b2 in patients with advanced neuromuscular disease (2). Although the patients with NMDs in this study had marked muscular atrophy and muscle had been replaced by fat, vascular tissue may be preserved even in atrophied muscle. Serum levels of antibody titers were found to be negatively correlated with residual muscle volume, but this correlation did not reach statistical significance. However, it is possible that this was due to the small number of patients in the NMDs group in this study. Previous report has not investigated the relationship between muscle remnants and increased antibody titers (2). The presence of a similar immune response to BNT162b2 indicates the non-muscle components as being more critical for immune response to mRNA vaccination. We tentatively conclude that, although the COVID-19 vaccine is administered to fat-replaced muscle, the COVID-19 vaccine may increase antibody titers sufficiently *via* preserved vascular tissue. Therefore, BNT162b2 appears to be effective in patients with NMDs.

### Safety of BNT162b2 in NMDs

In the present study, the frequency of any adverse reactions ≥ grade 1 was lower in the NMDs group than in the control group after each dose of the vaccine, but the frequency of any adverse reactions ≥grade 2 or 3 was comparable between the groups. This difference in the frequency of adverse reactions observed in each group may be attributed to the definition of the grade of adverse reactions. Grade 2 is defined as having symptoms and limitations in performing age-related instrumental activities of daily living. Because patients with NMDs did not perform many activities of daily living, they were possibly less informed about the symptoms of adverse reactions and did not have difficulties in their daily life. The lower frequency of analgesic use in the NMDs group after the second vaccination indicated that patients with NMDs did not have severe adverse reactions to vaccines. Therefore, BNT162b2 appears to be safe for use in patients with NMDs.

Previous research has found that several patients have developed bullous pemphigoid after vaccination with BNT162b2 (12). All five cases of bullous pemphigoid were found to have positive BP180 antibodies and developed symptoms after the second dose of vaccination. In this study, a patient with SMA developed bullous pemphigoid a few days after the first dose of BNT162b2. Therefore, the bullous pemphigoid in this patient was not considered to be an adverse reaction to BNT162b2 because it developed after the first dose of BNT162b2, rather than the second.

### Limitations of this study

This study was limited by the sample size of patients with NMDs, short follow-up period, and a lack of confirmation of the vaccination site. As the facility for NMDs and that for the vaccination were different, we could not confirm the vaccination site with ultrasound. Although it was reported that antibody titers after vaccination with BNT162b2 were significantly higher in females than in males (13), the gender impact on antibody titers was not evident in the NMDs group of this study. Larger studies with longer follow-ups are necessary to establish the efficacy and safety of BNT162b2 in this population.

## Conclusion

Antibody titers in patients with NMDs were similar to those of healthy controls and there was no difference in the percentage of adverse reactions to BNT162b2 between the NMDs group and healthy controls. Although BNT162b2 is administered by intramuscular injection, it appears to be effective and safe in patients with NMDs.

## Data availability statement

The raw data supporting the conclusions of this article will be made available by the authors, without undue reservation.

## Ethics statement

The studies involving human participants were reviewed and approved by the ethics committee of Aichi Medical University. Written informed consent to participate in this study was provided by the participants’ legal guardian/next of kin.

## Author contributions

HI contributed to the study design, interpretation of data, and was a major contributor in writing the manuscript. NI, KK, YM, YT, KM, AM, SK, TK, WO, and KN contributed to the acquisition and analysis of data from the patients. SH, MK, AO, and HI revised the manuscript critically for important intellectual content. All authors read and approved the final manuscript. All authors approved the final manuscript as submitted and agree to be accountable for all aspects of the work.

## Funding

The research of HI was supported by JSPS KAKENHI, Grant No. 21K07783. The research of AO was supported by JSPS KAKENHI, Grant No. 21K07810.

## Acknowledgments

We acknowledge the assistance of ENAGO with the English language editing.

## Conflict of interest

Author MK received royalties more than 1,000,000 yen from Takeda Pharmaceutical Company.

The remaining authors declare that the research was conducted in the absence of any commercial or financial relationships that could be construed as a potential conflict of interest.

## Publisher’s note

All claims expressed in this article are solely those of the authors and do not necessarily represent those of their affiliated organizations, or those of the publisher, the editors and the reviewers. Any product that may be evaluated in this article, or claim that may be made by its manufacturer, is not guaranteed or endorsed by the publisher.
